# A Comprehensive Study on the Internet of Underwater Things: Applications, Challenges, and Channel Models †

**DOI:** 10.3390/s17071477

**Published:** 2017-06-22

**Authors:** Chien-Chi Kao, Yi-Shan Lin, Geng-De Wu, Chun-Ju Huang

**Affiliations:** 1 Department of Communications, Navigation and Control Engineering, National Taiwan Ocean University, Keelung 20224, Taiwan; jerry810415@gmail.com (G.-D.W.); chuchu08311@gmail.com (C.-J.H.); 2 Department of Computer Science, National Chiao Tung University, Hsinchu 30010, Taiwan; yishanl@cs.nctu.edu.tw

**Keywords:** Internet of Things (IoT), Internet of Underwater Things (IoUT), smart city, Underwater Wireless Sensor Networks (UWSN), wireless sensor networks (WSN)

## Abstract

The Internet of Underwater Things (IoUT) is a novel class of Internet of Things (IoT), and is defined as the network of smart interconnected underwater objects. IoUT is expected to enable various practical applications, such as environmental monitoring, underwater exploration, and disaster prevention. With these applications, IoUT is regarded as one of the potential technologies toward developing smart cities. To support the concept of IoUT, Underwater Wireless Sensor Networks (UWSNs) have emerged as a promising network system. UWSNs are different from the traditional Territorial Wireless Sensor Networks (TWSNs), and have several unique properties, such as long propagation delay, narrow bandwidth, and low reliability. These unique properties would be great challenges for IoUT. In this paper, we provide a comprehensive study of IoUT, and the main contributions of this paper are threefold: (1) we introduce and classify the practical underwater applications that can highlight the importance of IoUT; (2) we point out the differences between UWSNs and traditional TWSNs, and these differences are the main challenges for IoUT; and (3) we investigate and evaluate the channel models, which are the technical core for designing reliable communication protocols on IoUT.

## 1. Introduction

**Figure 1:**
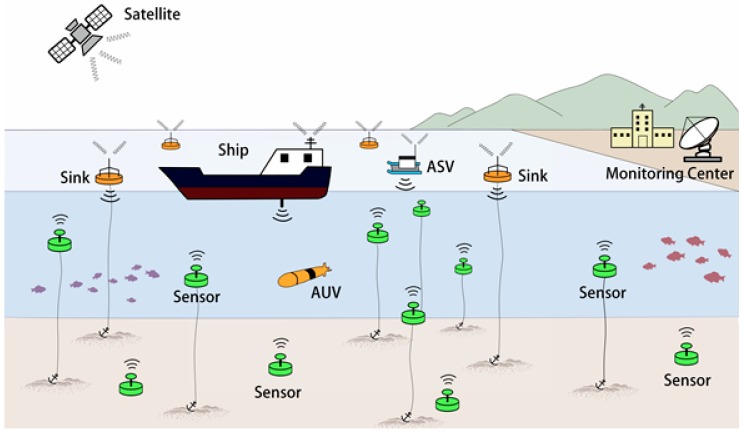
Network architecture of UWSNs.

In recent years, there has been a growing trend toward smart cities. One of the most important techniques to be used is the Internet of Things (IoT), which is defined as “the infrastructure of the information society” [1]. To the best of our knowledge, the concept of IoT was invented in 1985 [2]; then, in 2012, the Internet of Underwater Things (IoUT) was first discussed [3]. IoUT is defined as “the network of smart interconnected underwater objects”. As shown in Figure 1, the smart objects could be different types of underwater sensors, autonomous underwater vehicles (AUVs), autonomous surface vehicles (ASVs), buoys, ships, etc. IoUT is a new class of IoT, and an important part of smart city evolution.

In the literature, there have been several attempts ([3,4,5,6,7] and the references therein) to emphasize the importance of IoUT. We summarize the main reasons as follows. First, more than 70% of the earth’s surface is covered by water, and the majority of the underwater areas are still unexplored. Second, IoUT is expected to enable numerous applications in smart cities. Third, the underwater communication and equipment waterproofing technologies are at rather mature stage. Accordingly, this is an appropriate time to research IoUT.

For IoUT, Underwater Wireless Sensor Networks (UWSNs) have been regarded as a promising network system. Figure 1 shows the network architecture of UWSN. Note that readers interested in the UWSN equipment used in real-world projects are referred to [8,9,10,11]. As shown in Figure 1, UWSNs consist of several components. The main components are underwater sensors. In UWSNs, the sensors are the nodes with acoustic modems, and are distributed in either shallow or deep water. Each sensor node can sense (different sensors can sense different environmental information, such as the water quality, pressure, temperature, metal, and chemical and biological elements), relay, and forward data. The data should be transferred to the essential component(s) on the surface of the water, called sink(s). Sinks are the nodes with both acoustic and radio modems. Note that sinks can be buoys, ships, or ASVs. When data arrive at sinks (through acoustic channels), the sinks will forward data to the remote monitoring center (through radio channels). The monitoring center is often on the seashore, and is responsible for monitoring the water areas. The monitoring center collects, analyzes, and deals with the information from the water areas. In addition, the AUVs in the deep water can also be the optional components of a UWSN. The AUVs can help collect and forward data.

With the above useful and practical components, UWSNs are expected to enable a diverse range of IoUT applications, such as environmental monitoring, underwater exploration, and disaster prevention applications. However, the properties of UWSNs are different from those of the traditional Territorial Wireless Sensor Networks (TWSNs). The unique properties of UWSN, such as long propagation delay, narrow bandwidth, and low reliability, would be great challenges for IoUT. In the literature, many studies have concentrated on tackling the problems of the end-to-end delay, lack of bandwidth, and dynamic topology, but few studies have looked at the more fundamental problem of reliability. Note that low reliability would lead to frequent data retransmission, which would eventually result in longer delay, higher bandwidth consumption, and higher energy consumption. Therefore, reliability is of critical importance. To investigate the reliability of IoUT, the channel models play a vital role. In this paper, we thoroughly investigate the channel models, and validate the models through simulations.

This paper provides a comprehensive study of the IoUT applications, challenges, and channel models. The main contributions of this paper are threefold:

- we introduce and classify the potential IoUT applications;
- we point out the challenges for IoUT (i.e., the differences between UWSNs and TWSNs); and
- we investigate and evaluate the UWSN channel models, which are the technical core for IoUT.

The rest of the paper is organized as follows. Section 2 introduces the practical and potential IoUT applications, and classifies the applications into five types. Section 3 points out the challenges for IoUT, and discusses the challenges from seven aspects. Section 4 thoroughly investigates the channel models for IoUT. Section 5 presents the evaluation of the channel models through simulations. Then, we discuss how this work accelerates the pace of research on IoUT in Section 6. Section 7 provides the IoUT related work. Finally, Section 8 offers a conclusion.

## 2. Applications

**Figure 2:**
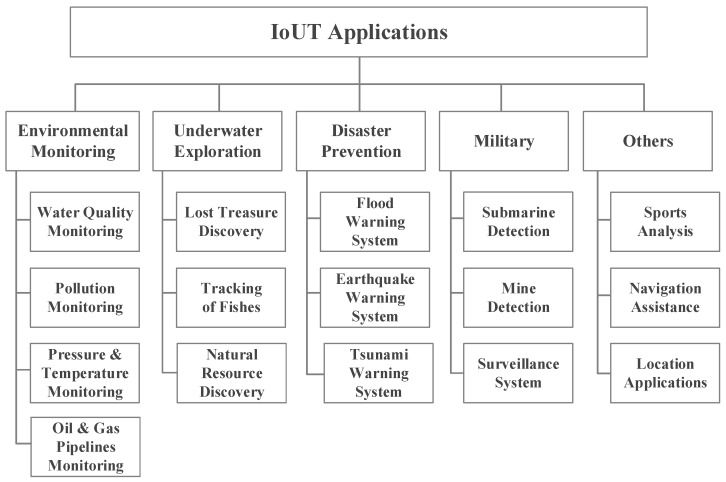
The IoUT applications.

In the last decade, researchers have presented numerous practical and potential IoUT applications [12,13,14,15,16,17,18,19,20,21,22,23,24,25,26,27,28,29]. We classify the applications into five types: (1) environmental monitoring; (2) underwater exploration; (3) disaster prevention; (4) military; and (5) others (Figure 2).

### 2.1. Environmental Monitoring

One of the most commonly used IoUT application types is environmental monitoring [12], including water quality monitoring [15], chemical and biological pollution monitoring [28], thermal pollution monitoring, pressure monitoring, and temperature monitoring [29]. In addition, oil and gas pipelines monitoring [16] can also be achieved by using UWSNs. Note that the environmental monitoring applications/systems have become increasingly popular and are now in great demand for global smart cities.

- The authors in [15] provide a practical example of the water quality monitoring system in India. Based on wireless sensor networks, they propose a suitable network architecture for a river water quality monitoring system. The monitoring system can help in continuous and remote monitoring of the water quality in India. Moreover, this paper presents the technical design of a sensor node that can be used for monitoring the pH of water. Note that the pH of water is one of the main parameters that indicates the quality of water. The sensed pH value will be wirelessly transmitted to the base station by using Zigbee communications. The authors also mentioned that the next phase of the project will include water-conductivity sensors, dissolved-oxygen sensors, and temperature sensors (in addition to the pH sensors).
- In [28], focusing on ocean pollution prevention, the authors reported an ad-hoc wireless sensor network to monitor the ocean pollution. Compared with the traditional long-range underwater communication, this work concentrates on the short-range multi-hop communication. Short-range communication implies that the sensor network can reuse some of the acoustic bandwidth and also avoid many of the challenges of long-range communication. More importantly, the short-range acoustic modems are less expensive than long-range modems. In addition, the authors propose a synchronization protocol that can improve the Quality of Service (QoS) and effectively enhance the lifetime of the underwater sensor network.
- In [29], the report presents a monitoring system deployed in Queensland, Australia, for monitoring the underwater temperature and luminosity (brightness), i.e., the information necessary to derive the health status of the coralline barrier. Based on wireless sensor networks, the authors present a real-world practical framework. They designed and implemented all aspects of the environmental monitoring system, including the sensing activity, data storage, local transmission (from the sensor nodes to the sinks), remote transmission (from the sinks to the control center), and visualization. For the local transmission between the sensors and sinks, the authors propose a power-aware TDMA protocol that can guarantee the robustness and adaptability when the network topology changes.
- In [16], the oil-and-gas related applications are highlighted. The authors first point out that the rapid advancement in wireless communication technologies has created a room for improvement in all areas of industrial practices related to oil and gas processing. From this point of view, the authors investigate and propose an oil and gas pipeline monitoring solution. By using wireless sensor networks, the proposed solution can provide reliable monitoring of oil and gas pipelines. Specifically, the proposed system is capable of reporting pipeline health-related statistics over large areas.
- In [12], the authors introduce numerous underwater monitoring applications. The authors define underwater monitoring as a network of sensors deployed underwater to monitor the underwater environments, characteristics, properties, or any objects of interest. The authors classify the underwater monitoring applications into three different categories and discuss each of them. The three categories are: (i) water quality monitoring; (ii) habitat monitoring; and (iii) monitoring underwater explorations. Readers interested in these types of applications are referred to [12] for a more detailed description.

### 2.2. Underwater Exploration

The concept of IoUT can be applied to the underwater lost-treasure discovery. For instance, the discovery of the Titanic in 1985 (by the Woods Hole Oceanographic Institution) benefited from the use of autonomous underwater vehicles (AUVs) [13]. By using UWSN technologies, IoUT can also be applied to tracking of fishes [13]. Moreover, underwater natural-resource discovery, such as minerals [17], metals, corals, and coral reefs [18], can all benefit from the infrastructure of UWSNs as well.

- Davis and Chang present an overview of UWSNs in [13]. They provide a valuable survey of various UWSN architectures, and indicate how the architectures facilitate unmanned underwater exploration. As mentioned in this paper, a number of successful lost-treasure discoveries were made with the help of UWSNs.
- The objective of [17] is to develop advanced communication techniques for efficient real-time investigation of large uninhibited oceans. To explore the oceanic environment, the authors propose a large-scale system that uses UWSNs. In particular, to investigate the underwater resources, the proposed sensor modules are installed with video capturing devices. Accordingly, the proposed system can be used to discover and excavate the mineral resources underwater. The simulation results are encouraging as the proposed system is helpful in large-scale surveillance and exploration.
- Bainbridge et al. carried out a practical research [18] in Australia. The research reports on the lessons learned from two years of operation of UWSNs deployed at seven coral reefs along the Great Barrier Reef in north-eastern Australia. The authors emphasize that “cool technologies” should be used in an extremely careful way, and should never overshadow the basic process of collecting data; the key is to strike a balance between what actually works in an operational sense versus what is the latest and greatest.

### 2.3. Disaster Prevention

The disaster prevention applications are one of the most crucial IoUT applications. This is because these applications may save our lives. Water-based natural disasters are potentially dangerous. For example, the Japanese nuclear accident, Fukushima Daiichi nuclear disaster (on 11 March 2011), was initiated primarily by the tsunami following the Tohoku earthquake. To provide applications for prevention of such disasters, IoUT is widely expected to detect flood [19], earthquake [20], and tsunami [21] underwater, and to offer early warning services.

- In [19], Perez et al. introduce the design of a practical real-time measurement system for flood monitoring. They deployed a real-world monitoring network with several real-time measurement devices in Spain. The testing results show that the proposed monitoring system is energy efficient, and has the robust communication capability for long-term collection of water-level data in many locations.
- The authors in [20] emphasize that the development of the early warning generation system based on UWSNs would undoubtedly contribute to saving human beings. The warning generation system can help save human lives when a natural disaster, e.g., an earthquake, occurs. One of the main contributions of this paper is to point out the physical layer challenges in establishing a UWSN system for warning generation. The authors conclude that: (1) reliable communication; (2) low-power design; and (3) efficient resource management will remain the major challenges for the UWSN-based warning generation systems.
- In [21], the authors discuss a number of approaches for tsunami detection, and propose an efficient architecture for such applications. The proposed architecture uses seismic pressure sensors to predict tsunamis underwater, and to forward/relay the sensed information by using the directed diffusion routing protocol. The authors also discuss and describe the analysis and response mechanisms in this work.

### 2.4. Military

The military often reflects the ability of a country to defend itself against any form of attack, including underwater attack. IoUT is required for defense purposes, and can be applied to submarine detection [22], underwater mine detection [23], and underwater surveillance systems [24]. These applications have great potential for the future naval forces.

- For submarine detection, the authors in [22] investigate the design of the sensor deployment, and propose a scheme to optimize the monitoring coverage. By taking the water depth, transmission range, and attenuation into account, the proposed scheme uses Particle Swarm Optimization (PSO) to determine the positions of the sensor nodes. The authors provide both the mathematical analysis and NS3-based simulations to verify the performance of the proposed scheme in terms of the monitoring coverage and number of sensor nodes required.
- In [23], Khaledi et al. analyze the design of underwater mine detection systems. Specifically, they consider two sonar alternatives and five towing-vehicle alternatives (i.e., one underwater vehicle alternative, two surface vehicle alternatives, and two airborne vehicle alternatives). They use a computer model to simulate the process of detecting underwater mines, and test each of the design alternatives. The results show that the underwater vehicle alternative consumes the least amount of energy. They also take the safety, speed, fuel economy, and probability of detection into account, and provide the corresponding analysis to indicate the best alternative.
- In [24], the authors propose a novel UWSN architecture for underwater surveillance systems. In the proposed architecture, when sensor nodes are deployed, the sensor nodes are first placed in surface buoys; then, the sensor nodes are lowered to different depths. The depths are determined by the proposed scheme for maximizing the surveillance coverage. Moreover, each node is equipped with multiple different types (e.g., acoustic, magnetic, radiation and mechanical) of micro-sensors. Reading the data collected from the micro-sensors, the authors further present a data-mining-based scheme for classifying the submarines, mines, and divers.

### 2.5. Others

With the advancements in UWSNs, increasing number of IoUT applications are becoming attractive, such as sports [25], navigation [26], and localization [27] applications. Note that the localization applications are particularly challenging yet very valuable. Specifically, in the real world, identifying the location underwater is challenging because Global Positioning System (GPS) cannot work in underwater environments. Imagine, for example, underwater sensors can be used as location reference points, and thus can provide swimmers, divers, ships, and underwater vehicles with valuable location information.

- The authors in [25] provide a systematic review, focusing on the biomechanical analysis of swimming by using the inertial sensors underwater. As mentioned in this paper, in recent years, the swimming athletes, coaches, and sport scientists have been working harder and harder to achieve a “fraction of a second” improvement. The use of sensors plays an important role in supporting performance enhancement from a biomechanical point of view. This paper introduces numerous existing methods that use wearable inertial sensors (including accelerometers, gyroscopes, and magnetometers) to assess the biomechanics of swimming performance. The results indicate that the underwater inertial sensors are suitable and reliable tools for swimming biomechanical analyses.
- As highlighted in [26], for a variety of applications, the sensed data can only be interpreted meaningfully when referenced to the location of the sensors; for example, navigation is such an application. The authors propose a multi-stage AUV-aided localization scheme for UWSNs. They conducted simulations to evaluate the performance of the proposed scheme in terms of the localization coverage, accuracy, and communication costs. The results indicate that good performance can be achieved by properly choosing the communication range.
- In [27], the authors propose an Anchor-Free Localization Algorithm, called AFLA, for underwater environments. Most of the traditional underwater localization algorithms assume that special underwater nodes (with special equipment) or AUVs can be used as “anchor nodes” to help identify the location underwater. The proposed AFLA does not need information from the special anchor nodes. Instead, AFLA uses the adjacent relationship of normal sensor nodes to identify the location underwater. The simulation results indicate that AFLA can be used in both the static and dynamic UWSNs.

## 3. Challenges

The current pace of research on IoUT is slow owing to the challenges arising from the uniqueness of UWSNs. In other words, the main challenges for IoUT are the differences between TWSNs and UWSNs. In this section, we discuss the challenges for IoUT based on seven aspects: (1) transmission media; (2) propagation speed; (3) transmission range; (4) transmission rate; (5) difficulty to recharge; (6) mobility; and (7) reliability. We summarize the differences/challenges in Table 1, and explain each of them below.

1. Transmission Media: In TWSNs, radio waves are often used for communications. However, UWSNs usually rely on acoustic communications rather than radio communications. This is because radio signals would be absorbed by water very quickly. Unfortunately, the properties of the acoustic waves are very different from those of the radio waves; subsequently, many of the communications protocols applied to TWSNs cannot be directly applied to UWSNs. Accordingly, the transmission media is one of the main challenges for IoUT.
2. Propagation Speed: The propagation speed in UWSNs is around 200,000 times slower than that in TWSNs. Specifically, the propagation speed of TWSN radio channels is 300,000,000 m/s, while the propagation speed of UWSN acoustic channels is only around 1500 m/s. Thus, guaranteeing bounded end-to-end delay would be a challenging issue for IoUT.
3. Transmission Range: The transmission range of UWSNs could be ten times longer than that of TWSNs. In underwater environments, to avoid being absorbed by water, signals need to be transmitted by using low frequency. Lower frequency implies longer transmission range; and longer transmission range implies more possibilities that interferences and collisions happen during data transmission. Therefore, prevention of interferences and collisions is regarded as one of the challenges for IoUT.
4. Transmission Rate: Compared with radio communications in TWSNs, acoustic communications in UWSNs use a narrow bandwidth. Owing to the narrow bandwidth, the transmission rate in UWSNs is generally very low (approximately 10 kbps). Hence, bandwidth utilization is an important concern for IoUT.
5. Difficulty to Recharge: In UWSNs, underwater sensors are difficult to recharge because the sensors are deployed in the underwater areas. When we consider potential cost of recharging the batteries of the underwater sensors, it is clear that energy efficiency would be another important concern for IoUT.
6. Mobility: UWSNs are mobile WSNs by nature. When there are water currents, the UWSN sensors may move and suffer from dynamic network topology changes. It is a challenging task to deal with the dynamic changes for IoUT.
7. Reliability: The link reliability in UWSNs is typically unstable and low. The reliability of a link means the successful delivery ratio (successful delivery ratio is defined as the ratio of the number of data that have been successfully delivered to a receiver compared to the number of data that have been sent out by the sender) between a pair of sensor nodes. In UWSNs, the successful delivery ratio would be severely affected by the transmission loss (Transmission loss is defined as the accumulated decrease in intensity of a waveform energy (e.g., the sound wave energy in this paper) when a wave propagates outwards from a source (e.g., from a sensor node in this paper)) and environmental noises. Because signals would be absorbed by water in underwater environments, the transmission loss is one of the severe problems in UWSNs. Furthermore, the environmental noises in UWSNs are composed of several complicated factors, including turbulence, shipping, waves, etc. As a result, the reliability issue is one of the most challenging issues for IoUT.

In summary, the differences (between TWSNs and UWSNs) are the main challenges for IoUT. On the one hand, the differences cause many challenging issues for IoUT; on the other hand, the challenging issues attract research on IoUT. In recent years, researchers have discussed and tackled some of the challenging issues. Although extensive research has been done on the issues of the propagation delay [30,31,32], bandwidth utilization [33,34,35], and energy efficiency [36,37,38] (see more related works in [39,40,41,42,43]), few studies have focused on the more fundamental issue of link reliability. As we can imagine, low reliability would lead to frequent data retransmission; then, frequent retransmission will eventually result in longer delay, higher bandwidth consumption, and higher energy consumption. The above imagination reminds us that reliability is a fundamental basis for efficient data transmission and is of critical importance. Therefore, we are particularly interested in the issue of reliability. In the next section, we introduce how to estimate the reliabilities of links through the underwater channel models.

## 4. Channel Models

To calculate the reliability of links on IoUT, we investigate the channel models for underwater environments. The aim of the models is to calculate the successful delivery ratio of each link over UWSNs. The successful delivery ratio (between a pair of sensor nodes) is equivalent to the reliability (of a link), and is one of the most advantageous information for designing reliable communication protocols on IoUT.

The underwater channel models consist of several elements. We divide the complicated channel models into two main parts. In the first part, we investigate the relationship between the transmitter power and signal-to-noise ratio (SNR); then, in the second part, we further investigate the relationship between the SNR and successful delivery ratio. To sum up, the channel models can provide researchers with a systematic way to calculate the reliability (i.e., successful delivery ratio) of each link over IoUT.

### 4.1. Relationship between the Transmitter Power and SNR

The goal of the first part is to determine the relationship between the transmitter power and SNR. We first define the transmitter power as *P* and the SNR as *γ_dB_*. Note that the unit of SNR is dB in this paper. The SNR can be divided into four parts [44], and be expressed as follows:

(1) $$\gamma_{dB}=S_{level,dB}-T_{loss,dB}-N_{level,dB}+D_{index,dB},$$

where *S_level,dB_*, *T_loss,dB_*, *N_level,dB_*, and *D_index,dB_* are the source level, transmission loss, noise level, and directivity index, respectively. For the rest of the paper, we simplify the notation. Specifically, we replace *γ_dB_*, *S_level,dB_*, *T_loss,dB_*, *N_level,dB_*, and *D_index,dB_* with *γ*, *S_level_*, *T_loss_*, *N_level_*, and *D_index_*, respectively. Accordingly, the SNR can be expressed as follows:

(2) $$\gamma =S_{level}-T_{loss}-N_{level}+D_{index}$$

Note that the unit of each factor (i.e., *γ*, *S_level_*, *T_loss_*, *N_level_*, and *D_index_*) is dB.

First, the source level *S_level_* is defined as the effective level of sound. To calculate *S_level_*, we need to start from the relationship between *S_level_* and transmitted signal intensity *I* [45]. The relationship can be expressed by

(3) $$S_{level}=10\left[\log\left(I\right)-\log\left(0.67\times 10^{-18}\right)\right].$$

To calculate *I*, we also need the relationship between the transmitter power *P* and the transmitted signal intensity *I*. The relationship can be expressed as follows

(4) $$P=4\pi r^{2}\times I,$$

where *r* is the transmission range (i.e., the radius of an imaginary sphere), and the surface area of a sphere is: $A=4\pi r^{2}.$ Specifically, we consider that the sound source is omnidirectional, and its power is uniformly distributed over the surface area of an imaginary sphere of radius *r*. Note that the units of *P* and *r* are W and m, respectively. Using Equation (4), we can first compute the intensity *I* by

(5) $$I=\frac{P}{4\pi r^{2}}.$$

Using Equations (3) and (5), we can first compute the source level *S_level_* according to the transmitter power *P* and its range *r* as follows

(6) $$\begin{array}{cc} S_{level} & =10[\log\left(\frac{P}{4\pi r^{2}}\right)-\log\left(0.67\times 10^{-18}\right)]\\ & =10\left[\log\left(P\right)-\log\left(4\pi r^{2}\right)-\log\left(0.67\times 10^{-18}\right)\right]. \end{array}$$

Second, the transmission loss *T_loss_* is defined as a measure of the rate at which sound energy is lost. The underwater transmission loss *T_loss_* [46] over a distance *d* (in m) for a signal of frequency *f* (in kHz) can be derived by

(7) $$T_{loss}=20\log d+\alpha (f)\times d\times 10^{-3},$$

where *α*(*f*) is the absorption coefficient (of sound in seawater) in dB/km. To calculate *α*(*f*), we can use Thorp’s formula [47], as follows:

(8) $$a(f)=\frac{0.11f^{2}}{1+f^{2}}+\frac{44f^{2}}{4100+f^{2}}+2.75\times 10^{-4}f^{2}+0.003.$$

Note that 20logd in Equation (7) is an approximation used to describe how sound level decreases when a sound wave propagates away from a sound source (uniformly in all directions), and the approximation is called spherical spreading. For spherical spreading, the rate at which energy decreases can be obtained by using the signal intensity. Let *I_SS_* be the signal intensity of spherical spreading, and we have the source signal intensity *I*. By spherical spreading, the intensity decreases as the inverse square of the transmission distance *d*. Accordingly, the spherical spreading *SS* is usually expressed in decibels (dB) as follows:

(9) $$SS=-10\log\left(\frac{I_{SS}}{I}\right)=-10\log\left(\frac{1}{d^{2}}\right)=20\log d$$

Third, the noise level *N_level_* is affected by a number of sources, such as turbulence, shipping, and waves. In the real world, it is difficult to compute the exact value of the noise level. Fortunately, the author in [48] provides a practical approximation of the noise level in underwater environments. The approximation of *N_level_* is given by the following equation:

(10) $$N_{level}=50-18\log f.$$

Fourth, the directivity index *D_index_* in underwater environments can be set as 0. This is because the underwater hydrophones are often omnidirectional.

(11) $$D_{index}=0$$

Combining Equations (2), (6), (7), (10) and (11), we can model the relationship between the transmitter power *P* and SNR *γ*, as follows:

(12) $$\begin{array}{cc} \gamma = & 10\left[\log\left(P\right)-\log\left(4\pi r^{2}\right)-\log\left(0.67\times 10^{-18}\right)\right]\\ & -20\log d-\alpha \left(f\right)\times d\times 10^{-3}-50+18\log f, \end{array}$$

where *r*, *d*, *α*(*f*), and *f* are transmission range, transmission distance, absorption coefficient, and frequency (of signal), respectively.

Based on the relationship between the transmitter power *P* and SNR *γ* in Equation (12), we can actually compute the SNR by combining Equations (8) and (12), as follows:

(13) $$\begin{array}{cc} \gamma = & 10\left[\log\left(P\right)-\log\left(4\pi r^{2}\right)-\log\left(0.67\times 10^{-18}\right)\right]-20\log d\\ & -\left[\left(\frac{0.11f^{2}}{1+f^{2}}+\frac{44f^{2}}{4100+f^{2}}+2.75\times 10^{-4}f^{2}+0.003\right)\times d\times 10^{-3}\right]\\ & -50+18\log f. \end{array}$$

### 4.2. Relationship between the SNR and Successful Delivery Ratio

After computing the SNR (by using the models of the relationship between the transmitter power and SNR), we further investigate the models of the relationship between the SNR and successful delivery ratio. The goal of the second part (of our channel models) is to calculate the successful delivery ratio, that is, the reliability of links over IoUT.

To achieve the above goal, we need to choose the suitable models for underwater environments. First, for modulation scheme, we choose the BPSK modulation because BPSK is widely used in underwater acoustic sensor networks [49]. Second, for signal propagation, we choose the Rayleigh fading channel because Rayleigh fading is an appropriate model for the multipath effect in both the shallow and deep water [47]. According to the models we choose, the bit error rate (BER) (the bit error rate (BER) means the number of bit errors per unit time) of BPSK in a Rayleigh fading channel can be derived [50], as follows

(14) $$BER(\gamma )=\frac{1}{2}\left(1-\sqrt{\frac{10^{\gamma /10}}{1+10^{\gamma /10}}}\right),$$

where *BER*(*γ*) is the estimated number of bit errors (the number of bit errors means the number of received bits (of a data stream over a communication channel) that have been altered due to noise, interference, distortion, or bit synchronization errors) per unit time over a communication channel when the SNR is equal to *γ*.

Based on the error rate of a single bit given by Equation (14), we can then compute the successful delivery ratio of a single bit, as follows

(15) $$\begin{array}{cc} P_{successful}^{1}(\gamma ) & =1-BER\left(\gamma \right)\\ & =1-\frac{1}{2}\left(1-\sqrt{\frac{10^{\gamma /10}}{1+10^{\gamma /10}}}\right)\\ & =\frac{1}{2}+\frac{1}{2}\sqrt{\frac{10^{\gamma /10}}{1+10^{\gamma /10}}}, \end{array}$$

where $P_{successful}^{1}(\gamma )$ is defined as the probability that a bit is successfully delivered when the SNR is equal to *γ*. Following the definition, let $P_{successful}^{m}(\gamma )$ be the probability that a packet (a packet is a formatted unit of data supported by computer communications links) is successfully delivered when the SNR is equal to *γ* and the size of the packet is equal to *m* bits. Finally, given the SNR *γ*, the successful delivery ratio of a packet with size *m* bits can be calculated by the following equation:

(16) $$\begin{array}{cc} P_{successful}^{m}(\gamma ) & ={\left[1-BER\left(\gamma \right)\right]}^{m}\\ & ={\left[P_{successful}^{1}(\gamma )\right]}^{m}\\ & ={\left(\frac{1}{2}+\frac{1}{2}\sqrt{\frac{10^{\gamma /10}}{1+10^{\gamma /10}}}\right)}^{m}. \end{array}$$

Based on all of the channel models proposed in this section, given the transmitter power *P*, transmission range *r*, transmission distance *d*, and frequency *f*, we can first compute the SNR *γ* for IoUT by using the first part of the channel models, i.e., Equation (13). Given the SNR *γ*, we can then calculate the bit error rate *BER*(*γ*) for IoUT by using the second part of the channel models, i.e., Equation (14). Finally, we compute the successful delivery ratio of any pair of sensor nodes using Equation (16). The delivery ratio represents the probability that a data packet can be successfully delivered from a sender to a receiver, and stands for the reliability of an underwater wireless link. In summary, given the transmitter power, the channel models can provide researchers with a quick and accurate way of estimating the SNR, BER, and successful delivery ratio, that is, the reliability for UWSNs/IoUT.

### 4.3. Example

We provide a specific example here to demonstrate how the channel models work. Note that we will also justify the channel models for a wider range of parameters by conducting simulations in Section 5. In the following example, the SNR, BER, and successful delivery ratio are computed.

First, we set the transmitter power, transmission range/distance, and frequency as 2 W, 300 m, and 10 kHz, respectively. When the transmitter power is 2 W, the source level *S_level_* can be computed by Equation (6):

$$S_{level}=10\left[\log\left(2\right)-\log\left(4\pi \times 300^{2}\right)-\log\left(0.67\times 10^{-18}\right)\right]\approx 124\;\mathrm{dB}$$

Second, given the transmission distance (300 m) and frequency (10 kHz), the transmission loss *T_loss_* can be computed by Equation (7):

$$T_{loss}=20\log\left(300\right)+\alpha (10)\times 300\times 10^{-3}\approx 50\;\mathrm{dB}$$

Third, by substituting the frequency (10 kHz) into Equation (10), the noise level *N_level_* is roughly:

$$N_{level}=50-18\log\left(10\right)=32\;\mathrm{dB}$$

Combining all of the above results and Equation (2), i.e., the combination is equivalent to Equation (13), we can compute the SNR as follows:

γ=124−50−32+0=42 dB

Then, substituting the SNR into Equation (14), we can compute the bit error rate *BER*(*γ*):

$$BER(\gamma )=\frac{1}{2}\left(1-\sqrt{\frac{10^{42/10}}{1+10^{42/10}}}\right)\approx 1.58\times 10^{-5}$$

Finally, for a packet with size 3 KB, the successful delivery ratio can be computed by using Equation (16):

$$P_{successful}^{m}(\gamma )={\left[1-\left(1.58\times 10^{-5}\right)\right]}^{3\times 1024\times 8}\approx 0.6782$$

The successful delivery ratio is about 67.82%. The numerical example examines the equations of the channel models step-by-step for a specific case. For more cases (and more discussion), we conducted the simulations in Section 5.

## 5. Results

In this section, we validated the UWSN channel models through simulations. Note that the UWSN channel models are the technical core for IoUT.

### 5.1. Simulation Environment

The simulations were conducted by using C++. We consider an underwater wireless sensor network (UWSN) with the BPSK modulation [49] and Rayleigh fading channel [47]. We set the simulation parameters according to the commercial LinkQuest underwater acoustic modems [51]; specifically, we set the transmitter powers in the range of 1–40 W [52]. For UWSN communications, we set the frequency and packet size as 10 KHz [53] and 3 KB [54], respectively.

The output measures are (1) SNR; (2) BER; and (3) delivery ratio. As mentioned in Section 4, the proposed channel models can produce the SNR, BER, and delivery ratio sequentially. First, the channel models calculate the four main elements of the SNR (i.e., the source level, transmission loss, noise level, and directivity index), and thus produce the SNR. Then, after choosing the appropriate models (i.e., the BPSK modulation and Rayleigh fading channel) according to the underwater characteristics, the channel models produce the BER. Finally, considering the practical packet size, the channel models produce the delivery ratio (i.e., the reliability). To validate the whole channel model, we evaluate each part of the models as follows.

### 5.2. Evaluation of the Signal-to-Noise Ratio (SNR)

**Figure 3:**
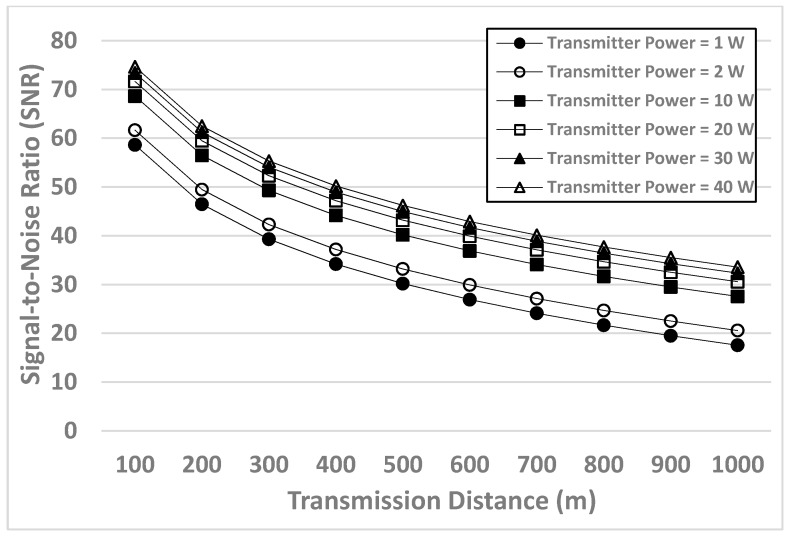
Simulation results of the Signal-to-Noise Ratio (SNR) when the channel models are applied to different transmitter powers (1–40 W) and distances (100–1000 m).

Figure 3 shows the simulation results of the SNR when the transmitter powers are set as 1 W, 2 W, 10 W, 20 W, 30 W, and 40 W. This figure plots the SNR as a function of the transmission distance. The distances are from 100 m to 1000 m.

First, when the transmission distance increases, the SNR decreases. This is because the “transmission loss” is one of the main elements of the SNR. When the distance increases, the transmission loss would be accumulated; subsequently, the accumulated transmission loss makes a negative impact on the SNR. Therefore, the SNR is inversely proportional to the transmission distance between the underwater sensor nodes. The same phenomenon occurs in all the cases of different transmitter powers. The phenomenon confirms that our channel models are reasonable (when calculating the SNR) and applicable to different transmitter powers.

Second, when the transmitter power is higher, the SNR is higher. Specifically, when the transmission distance is the same, the transmitter powers from low SNR to high SNR are: 1 W, 2 W, 10 W, 20 W, 30 W, and then 40 W. This is because one of the main elements of the SNR is the “source level”; when the transmitter power is higher, the source level is higher. Thereby, the SNR is directly proportional to the transmitter power. Because the results are consistent in all the cases of different transmission distances (from 100 m to 1000 m), the simulation results confirm that our channel models are also applicable to different distances.

### 5.3. Evaluation of the Bit Error Rate (BER)

**Figure 4:**
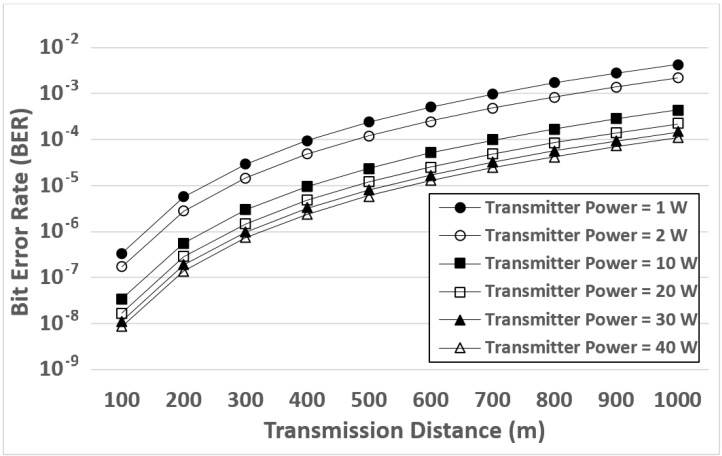
Simulation results of the Bit Error Rate (BER) when the channel models are applied to different transmitter powers (1–40 W) and distances (100–1000 m).

Figure 4 shows the simulation results of the BER. This figure plots the BER as a function of the transmission distance. The transmission distances are from 100 m to 1000 m, and the transmitter powers are set as 1 W, 2 W, 10 W, 20 W, 30 W, and 40 W.

When the transmission distance increases, the BER also increases. This is because when the distance increases, the SNR decreases (as shown in Figure 3). For wireless communications, the lower SNR would lead to the higher BER. To sum up, when the distance increases, the SNR decreases, and thereby the BER increases. As shown in Figure 4, the same phenomenon occurs in all the cases of different transmitter powers. Because the phenomenon remains consistent, it confirms that when calculating the BER, our channel models are reasonable and applicable to different transmitter powers.

In addition, when the transmitter power is higher, the BER is lower. Specifically, when the transmission distance is the same, the transmitter powers from high BERs to low BERs are: 1 W, 2 W, 10 W, 20 W, 30 W, and then 40 W. We can explain the phenomenon by using the relationship between the SNR and BER. When the transmitter power is higher, the SNR is higher (as shown in Figure 3); when the SNR is higher, the BER should be lower (as mentioned in the above paragraph). As a result, the BER is inversely proportional to the transmitter power (i.e., when the transmitter power is higher, the BER is lower). Because the results are consistent in all the cases of different transmission distances (from 100 m to 1000 m), the simulation results confirm that when calculating the BER, our channel models are also applicable to different distances.

Note that although the 10 W, 20 W, 30 W and 40 W transmitters outperform the 1 W and 2 W transmitters in terms of the BER, the 10 W, 20 W, 30 W, and 40 W devices are potentially heavier and more expensive [52]. Our simulation results indicate the trade-off between performance and cost. Specifically, the 1 W and 2 W transmitters may cause higher BER (lower performance) for underwater communications, whereas the 1 W and 2 W transmitters are lighter and less expensive.

### 5.4. Evaluation of the Successful Delivery Ratio (Reliability)

**Figure 5:**
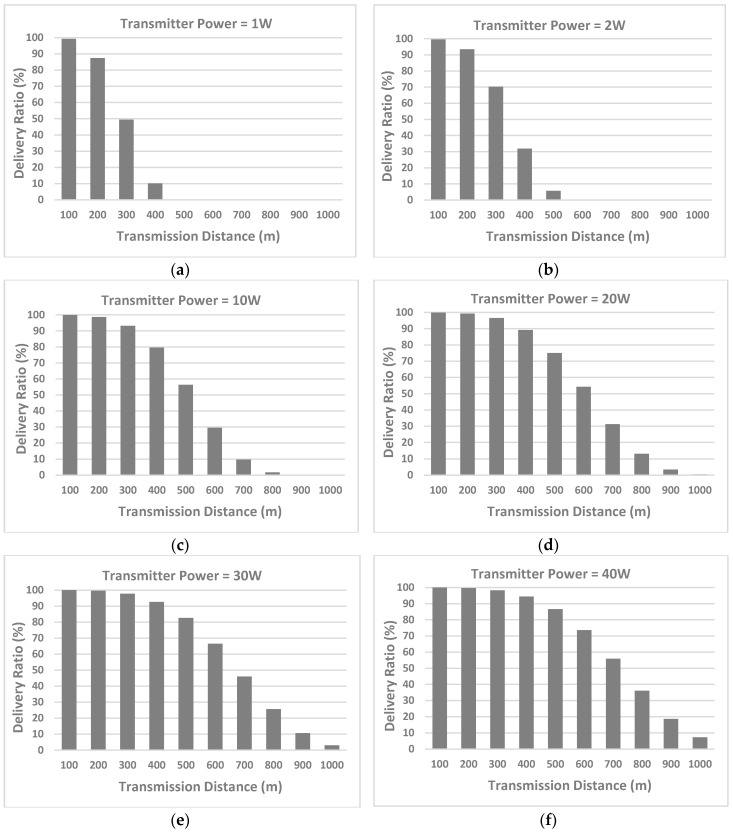
Simulation results of the successful delivery ratio when the channel models are applied to different transmitter powers (1–40 W) and distances (100–1000 m) (**a**) Transmitter power = 1 W; (**b**) Transmitter power = 2 W; (**c**) Transmitter power = 10 W; (**d**) Transmitter power = 20 W; (**e**) Transmitter power = 30 W; (**f**) Transmitter power = 40 W.

Figure 5 shows the simulation results of the successful delivery ratio, that is, the reliability for underwater wireless communications. To thoroughly investigate the reliability of UWSNs, we provide the detailed simulation results in Figure 5a–f. Specifically, Figure 5a–f are the results when the transmitter powers are set as 1 W, 2 W, 10 W, 20 W, 30 W, and 40 W, respectively. Each of the figures provides the delivery ratio when the transmission distances are equal to 100 m, 200 m, 300 m, 400 m, 500 m, 600 m, 700 m, 800 m, 900 m, and 1000 m.

In all figures, we can see that, when the transmission distance increases, the successful delivery ratio decreases. We explain this phenomenon as follows. When the transmission distance increases, the SNR decreases (as shown in Figure 3), and thus, the BER increases (as shown in Figure 4); more BER means more number of received bits that have been altered owing to noise, interference, distortion, or bit synchronization errors. Accordingly, when transmitting a whole packet in UWSNs, more BER would result in less successful delivery ratio. In summary, when the distance increases, the SNR decreases, the BER increases, and thereby the delivery ratio decreases. The phenomenon remains consistent in all the cases of different transmitter powers (i.e., 1 W, 2 W, 10 W, 20 W, 30 W, and 40 W) in Figure 5a–f. The simulation results confirm that our channel models are reasonable in each step (from the SNR, the BER, to the delivery ratio), and are applicable to different transmitter powers.

When the transmitter power increases from Figure 5a–f, we can find that the successful delivery ratio increases gradually. We take the transmission distance of 500 m as an example. For 500 m, when the transmitter powers are 1 W, 2 W, 10 W, 20 W, 30 W and 40 W, the corresponding delivery ratios are about 0%, 5%, 57%, 75%, 82%, and 88%, respectively, as shown in Figure 5a–f. The explanation is as follows. When the transmitter power is higher, the SNR is higher (as shown in Figure 3), and thereby the BER is lower (as shown in Figure 4); lower BER means lower probability of being wrong during packet transmissions. In other words, lower BER means higher successful probability of delivering packets. Accordingly, higher transmitter power produces higher SNR, and lower BER, which eventually results in higher successful delivery ratio. Note that the phenomenon occurs not only in the case of 500 m, but also in all the cases of different distances. Because the results are consistent in all the cases, the simulation results confirm that when calculating the successful delivery ratio (i.e., the reliability), our channel models are applicable to different distances.

## 6. Discussion

In this section, we discuss how this work accelerates the pace of research on IoUT. In Section 2, we first introduced numerous potential IoUT applications that are attractive to researchers. Then, in Section 3, we pointed out that a number of challenges exist. The challenging issues are attractive to researchers as well. Because reliability is one of the major concerns of IoUT, we investigated the UWSN channel models to calculate the reliability for IoUT in Section 4. The channel models provide a systematic way to compute the reliability between a pair of sensor nodes over IoUT. In Section 5, we conducted simulations to validate each part of the channel models, and confirmed that the models are applicable to underwater environments. We expect that the efforts would add value to the future research on IoUT.

**Figure 6:**
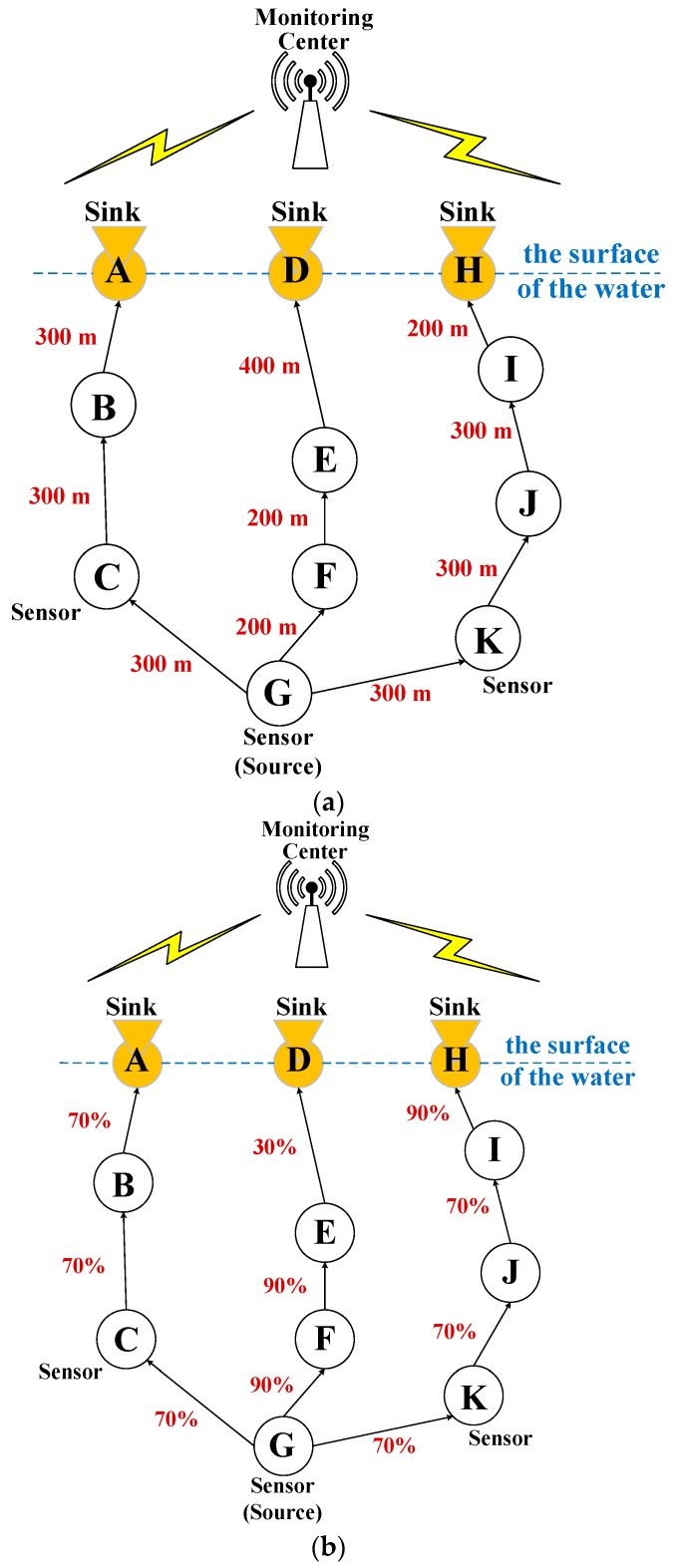
Example of the routing paths in UWSNs with: (**a**) the information of link distance; and (**b**) the information of link reliability.

Following the previous sections, this section provides an extra example to demonstrate how the channel models help researchers investigate the design of the reliable IoUT communication protocols as follows. We take the IoUT routing protocol as an example. By definition, a routing protocol needs to specify how to select routes (i.e., paths) to forward data in communication networks. Accordingly, an IoUT routing protocol needs to select the appropriate paths to forward data from a source sensor node to a sink in UWSNs. A delay-sensitive routing protocol may select the shortest path for data transmission. However, for wireless communications, the shortest path is often different from the most reliable path. Figure 6 shows a typical example. In Figure 6a, the shortest path from the source sensor node (i.e., node G) to the sink (i.e., any sink on the surface of the water) is: node G, node F, node E, and then sink D. The distance of the shortest path is: 200 m + 200 m + 400 m = 800 m. However, as shown in Figure 6b, the most reliable path in the same UWSN is a different path: node G, node C, node B, and then sink A. This is because the reliability of the most reliable path is: 70% × 70% × 70% = 34.3%. This is one simple example to point out the importance of the reliability between a pair of sensor nodes in UWSNs. Without the information of the link reliability, it would be very difficult for a routing protocol to select the most reliable path. Using reasonable underwater channel models, researchers can calculate the reliabilities of links (as described in Section 4), and thereby design a reliable routing or communication protocol for IoUT.

In addition, after studying many IoUT related works, we find that some of the early related works did not fully take the practical applications, challenges, and channel models into account. For instance, some of the related works evaluated the performance of the proposed IoUT communication protocols by using the traditional territorial channel models. By replacing the territorial channel models with the practical underwater channel models, the researchers may discover new issues regarding the existing IoUT protocols. From this point of view, we believe that this work would assist some existing IoUT protocols to be refined.

## 7. Related Work

In this section, we introduce three state-of-the-art IoUT-related works. These works can provide more information of IoUT, and offer the potential research directions in the near feature.

- In [4], the research effort has focused on the MAC-layer packet scheduling for IoUT. Considering the design challenges of MAC protocols, the authors presented an Energy-Aware scheduling with Spatial-Temporal reuse (EAST) protocol for IoUT. The authors mentioned the design challenges of the IoUT MAC protocols, including: (1) sensor network lifetime; (2) acoustic channel utilization; and (3) packet loss problems. EAST utilizes the information of data buffer and traffic load to switch the energy modes (i.e., the listen and sleep mode) of underwater sensor nodes, and thereby achieves both high energy efficiency and high channel utilization.
- In [5], the authors concentrated on the network-layer routing protocol for IoUT. Considering the IoUT challenges of data transmission and of energy efficiency, the authors proposed an Enhanced Channel-Aware Routing Protocol (E-CARP) for IoUT. E-CARP exploits the greedy strategy to deliver packets hop-by-hop. Specifically, E-CARP simplifies the relay-node selection method and reduces control packets, and thereby reduces the communication cost and energy consumption. As a result, E-CARP can reserve energy and lengthen the lifetime of the underwater sensor system for IoUT.
- In [6], the study focused on the design and implementation of the smart-city IoUT system. The authors proposed the Smart Environment Monitoring and Analytics in Real-time system (SEMAR). SEMAR is a combination of water quality monitoring system, coral reef monitoring system, wireless mesh network system, and Big Data system. SEMAR collects data by using the underwater sensors, remotely operated vehicles (ROVs), and underwater cameras. The collected data would be delivered to the monitoring center. For data processing (in the monitoring center), the authors point out that using Hadoop Distributed File Systems (HDFS) can perform better than using SQL systems, especially in the cases of processing large/huge amounts of data.

## 8. Conclusions

In this paper, we study a new class of IoT, called IoUT, i.e., the Internet of Underwater Things. This paper provides useful information about the IoUT: (1) applications; (2) challenges; and (3) channel models. The simulation results have confirmed that our channel models are practical and applicable to different transmitter powers (i.e., 1–40 W) and to different transmission distances (i.e., 100–1000 m). Specifically, when the transmitter power is higher, the corresponding SNR is higher, the BER is lower, and thereby the successful delivery ratio is higher. Furthermore, when the transmission distance increases, the corresponding SNR decreases, and the BER increases, the delivery ratio decreases. The channel models are reasonable, and are expected to help researchers investigate the communication protocols on IoUT. In the future, we will also apply the channel models to further investigate the design of different IoUT communication protocols, such as the MAC protocols and routing protocols.

## Figures and Tables

**Table 1:** The differences between TWSNs and UWSNs.

Features	TWSNs	UWSNs
Transmission Media	Radio Wave	Sound Wave
Propagation Speed	300,000,000 m/s	1500 m/s
Transmission Range	10 m–100 m	100 m–10,000 m
Transmission Rate	~250 kbps	~10 kbps
Difficulty to Recharge	Depend on Applications	Difficult
Mobility (of Nodes)	Depend on Applications	High
Reliability (of Links)	Depend on Applications	Low

## References

[1] ITU-T IoT-GSI Internet of Things Global Standards Initiative. http://www.itu.int/en/ITU-T/gsi/iot/Pages/default.aspx.

[2] Sharma C. Correcting the IoT History. http://www.chetansharma.com/correcting-the-iot-history/.

[3] Domingo M.C. (2012). An overview of the internet of underwater things. J. Netw. Comput. Appl..

[4] Xu M., Liu L. (2016). Sender-receiver role-based energy-aware scheduling for Internet of Underwater Things. IEEE Trans. Emerg. Top. Comput..

[5] Zhou Z., Yao B., Xing R., Shu L., Bu S. (2016). E-CARP: An energy efficient routing protocol for UWSNs in the internet of underwater things. IEEE Sens. J..

[6] Berlian M.H., Sahputra T.E.R., Ardi B.J.W., Dzatmika L.W., Besari A.R.A., Sudibyo R.W., Sukaridhoto S. Design and implementation of smart environment monitoring and analytics in real-time system framework based on internet of underwater things and big data. Proceedings of the IEEE International Electronics Symposium (IES).

[7] Fang S., Xu L.D., Zhu Y., Ahati J., Pei H., Yan J., Liu Z. (2014). An integrated system for regional environmental monitoring and management based on internet of things. IEEE Trans. Ind. Inform..

[8] Petrioli C., Petroccia R., Potter J.R., Spaccini D. (2015). The SUNSET framework for simulation, emulation and at-sea testing of underwater wireless sensor networks. Ad Hoc Netw..

[9] Martins R., Sousa J.B., Caldas R., Petrioli C., Potter J. SUNRISE project: Porto university testbed. Proceedings of the IEEE Underwater Communications and Networking (UComms).

[10] Petrioli C., Petroccia R. SUNSET: Simulation, emulation and real-life testing of underwater wireless sensor networks. Proceedings of the IEEE Underwater Communications and Networking (UComms).

[11] Petrioli C., Petroccia R., Spaccini D. SUNSET version 2.0: Enhanced framework for simulation, emulation and real-life testing of underwater wireless sensor networks. Proceedings of the ACM International Conference on Underwater Networks and Systems (WUWNet).

[12] Felemban E., Shaikh F.K., Qureshi U.M., Sheikh A.A., Qaisar S.B. (2015). Underwater sensor network applications: A comprehensive survey. Int. J. Distrib. Sens. Netw..

[13] Davis A., Chang H. Underwater wireless sensor networks. Proceedings of the IEEE OCEANS.

[14] Lloret J. (2013). Underwater sensor nodes and networks. Sensors.

[15] Menon K.A.U., Divya P., Ramesh M.V. Wireless sensor network for river water quality monitoring in India. Proceedings of the IEEE International Conference on Computing, Communication and Networking Technologies (ICCCNT).

[16] Saeed H., Ali S., Rashid S., Qaisar S., Felemban E. Reliable monitoring of oil and gas pipelines using wireless sensor network (WSN): REMONG. Proceedings of the IEEE International Conference on System of Systems Engineering (SOSE).

[17] Srinivas S., Ranjitha P., Ramya R., Narendra K.G. Investigation of oceanic environment using large-scale UWSN and UANETs. Proceedings of the IEEE International Conference on Wireless Communications, Networking and Mobile Computing (WiCOM).

[18] Bainbridge S., Eggeling D., Page G. (2011). Lessons from the field—Two years of deploying operational wireless sensor networks on the great barrier reef. Sensors.

[19] Perez R.M., Pintado J.G., Gómez A.S. (2012). A real-time measurement system for long-life flood monitoring and warning applications. Sensors.

[20] Kumar P., Kumar P., Priyadarshini P., Srija Underwater acoustic sensor network for early warning generation. Proceedings of the IEEE OCEANS.

[21] Casey K., Lim A., Dozier G. (2008). A sensor network architecture for tsunami detection and response. Int. J. Distrib. Sens. Netw..

[22] Manjula R.B., Manvi S.S. Coverage optimization based sensor deployment by using PSO for anti-submarine detection in UWASNs. Proceedings of the International Symposium on Ocean Electronics.

[23] Khaledi S., Mann H., Perkovich J., Zayed S. Design of an underwater mine detection system. Proceedings of the IEEE Systems and Information Engineering Design Symposium (SIEDS).

[24] Cayirci E., Tezcan H., Dogan Y., Coskun V. (2006). Wireless sensor networks for underwater surveillance systems. Ad Hoc Netw..

[25] Magalhaes F.A., Vannozzi G., Gatta G., Fantozzi S. (2015). Wearable inertial sensors in swimming motion analysis: A systematic review. J. Sports Sci..

[26] Waldmeyer M., Tan H.P., Seah W.K.G. Multi-stage AUV-aided localization for underwater wireless sensor networks. Proceedings of the IEEE Workshops of Advanced Information Networking and Applications (WAINA).

[27] Guo Y., Liu Y. (2013). Localization for anchor-free underwater sensor networks. Comput. Electr. Eng..

[28] Khan A., Jenkins L. Undersea wireless sensor network for ocean pollution prevention. Proceedings of the IEEE International Conference on Communication Systems Software and Middleware (COMSWARE).

[29] Alippi C., Camplani R., Galperti C., Roveri M. (2011). A robust adaptive solar-powered WSN framework for aquatic environmental monitoring. IEEE Sens. J..

[30] Shyan C.Y., Ying J.T., Wei L.Y., Che T.I. (2010). A low propagation delay multi-path routing protocol for underwater sensor networks. J. Int. Technol..

[31] Noh Y., Lee U., Han S., Wang P., Torres D., Kim J., Gerla M. (2014). DOTS: A propagation delay-aware opportunistic MAC protocol for mobile underwater networks. IEEE Trans. Mob. Comput..

[32] Gao C., Liu Z., Cao B., Mu L. Relay selection scheme based on propagation delay for cooperative underwater acoustic network. Proceedings of the IEEE International Conference on Wireless Communications and Signal Processing (WCSP).

[33] Chen Y.D., Liu S.S., Chang C.M., Shih K.P. CS-MAC: A Channel Stealing MAC protocol for improving bandwidth utilization in underwater wireless acoustic networks. Proceedings of the IEEE OCEANS.

[34] Liao Z., Li D., Chen J. (2015). Joint bandwidth optimization and media access control for multihop underwater acoustic sensor networks. IEEE Sens. J..

[35] Fahim H., Javaid N., Qasim U., Khan Z.A., Javed S., Hayat A., Iqbal Z., Rehman G. Interference and bandwidth aware depth based routing protocols in underwater WSNs. Proceedings of the IEEE International Conference on Innovative Mobile and Internet Services in Ubiquitous Computing (IMIS).

[36] Xu J., Li K., Min G., Lin K., Qu W. (2012). Energy-efficient tree-based multipath power control for underwater sensor networks. IEEE Trans. Parallel Distrib. Syst..

[37] Wahid A., Lee S., Kim D. (2014). A reliable and energy-efficient routing protocol for underwater wireless sensor networks. Int. J. Commun. Syst..

[38] Shah M., Javaid N., Tariq S., Imran M., Alnuem M. A balanced energy consumption protocol for underwater ASNs. Proceedings of the IEEE International Conference on Network-Based Information Systems (NBIS).

[39] Climent S., Sanchez A., Capella J.V., Meratnia N., Serrano J.J. (2014). Underwater acoustic wireless sensor networks: Advances and future trends in physical, MAC and routing layers. Sensors.

[40] Darehshoorzadeh A., Boukerche A. (2015). Underwater sensor networks: A new challenge for opportunistic routing protocols. IEEE Commun. Mag..

[41] Han G., Jiang J., Bao N., Wan L., Guizani M. (2015). Routing protocols for underwater wireless sensor networks. IEEE Commun. Mag..

[42] Chen K., Ma M., Cheng E., Yuan F., Su W. (2014). A survey on MAC protocols for underwater wireless sensor networks. IEEE Commun. Surv. Tutor..

[43] Menon V.G., Prathap J.P.M. Comparative Analysis of Opportunistic Routing Protocols for Underwater Acoustic Sensor Networks. Proceedings of the IEEE International Conference on Emerging Technological Trends (ICETT).

[44] Jurdak R., Lopes C.V., Baldi P. (2004). Battery lifetime estimation and optimization for underwater sensor networks. IEEE Sens. Netw. Oper..

[45] National Physical Laboratory (NPL) Underwater Acoustics Concepts—Source Levels (SL). http://resource.npl.co.uk/acoustics/techguides/concepts/sl.html.

[46] Forming the Acoustic Equations. http://www.fao.org/docrep/X5818E/x5818e05.htm.

[47] Yang H., Liu B., Ren F., Wen H., Lin C. Optimization of energy efficient transmission in underwater sensor networks. Proceedings of the IEEE Global Telecommunications Conference (GLOBECOM).

[48] Stojanovic M. On the relationship between capacity and distance in an underwater acoustic communication channel. Proceedings of the ACM International Workshop on Underwater Networks (WUWNet).

[49] Noh Y., Lee U., Wang P. Pressure routing for underwater sensor networks. Proceedings of the IEEE INFOCOM.

[50] Goldsmith A. (2005). Wireless Communications.

[51] SoundLink Underwater Acoustic Modems. http://www.link-quest.com/.

[52] Xu J., Li K., Min G. (2012). Reliable and energy-efficient multipath communications in underwater sensor networks. IEEE Trans. Parallel Distrib. Syst..

[53] Jiang J., Han G., Guo H., Shu L., Rodrigues J.J.P.C. (2016). Geographic multipath routing based on geospatial division in duty-cycled under water wireless sensor networks. J. Netw. Comput. Appl..

[54] Basagni S., Petrioli C., Petroccia R., Stojanovic M. (2012). Optimized Packet Size Selection in Underwater Wireless Sensor Network Communications. IEEE J. Ocean. Eng..

